# First Report of Cactus Weevil Feeding on *Selenicereus undatus* in Jalisco, Mexico

**DOI:** 10.3390/plants14081162

**Published:** 2025-04-09

**Authors:** Haidel Vargas-Madriz, Citlally Topete-Corona, Ausencio Azuara-Dominguez, Ángel Félix Vargas-Madriz, Martha Olivia Lazaro-Dzul, Jesús Alberto Acuña-Soto, Aarón Kuri-García, Crystian Sadiel Venegas-Barrera

**Affiliations:** 1 Department of Agricultural Production, University Center of the South Coast, University of Guadalajara, Avenida Independencia Nacional 151, Colonia Centro, Autlán de Navarro 48900, Mexico; haidel.vargas@academicos.udg.mx; 2 Graduate Program in Biology, National Technological Institute of Mexico, Ciudad Victoria Institute of Technology, Boulevard Emilio Portes Gil No. 1301, Ciudad Victoria 87010, Mexico; dzulmartha@gmail.com (M.O.L.-D.); crystian.vb@cdvictoria.tecnm.mx (C.S.V.-B.); 3 Laboratory of Cellular and Molecular Biology, Faculty of Natural Sciences, Autonomous University of Querétaro, Querétaro 76230, Mexico; angel.vargas@uaq.mx (Á.F.V.-M.); aaron.kuri@uaq.mx (A.K.-G.); 4 Division of Agricultural Innovation Engineering, TecNM—Technological Institute of Tlatlauquitepec, Tlatlauquitepec 73680, Mexico; coleoptero77@hotmail.com

**Keywords:** México, *Cactophagus spinolae*, *Selenicereus undatus*, agricultural pests, cacti damage, phytosanitary management

## Abstract

The cactus *Selenicereus undatus* (*S. undatus*), known as pitahaya or dragon fruit, is one of the pitaya species of economic importance for different countries worldwide, particularly in China, Mexico, Australia, Vietnam, Indonesia, and the United States, among other countries. On the other hand, *Cactophagus spinolae* (*C. spinolae*) is a pest insect found in Mexico, known as the cactus weevil or borer. This study reports, for the first time, *C. spinolae* feeding and damage to the vascular stem and flower bud of wild *S. undatus* plants in Jalisco, Mexico. Field sampling was conducted in wild populations of *S. undatus* between July and October 2024. The results of our study confirm that *C. spinolae* actively feeds on *S. undatus*, causing damage to the plant, primarily to the vascular cylinder of mature and immature stems, as well as to flower buds and tissues. During monitoring, we observed a total of nine eggs, nine larvae, and sixteen adults in different plant samples, confirming that S. undatus serves as a reproductive host for *C. spinolae*. The results highlight the threat of *C. spinolae* to *S. undatus* in the State of Jalisco, which highlights the need to incorporate new agricultural strategies to mitigate the impact that this insect has on pitahaya.

## 1. Introduction

*Selenicereus undatus* (*S. undatus*) is a cactus native to Mexico and South-Central America [1,2], recognized worldwide for the nutritional and commercial value of its fruit called “pitahaya” or “dragon fruit”, which is mainly produced by several countries, such as China, Mexico, Australia, Vietnam, Indonesia, and the United States, among other places [3]. Some studies mention that the commercialization of *S. undatus* fruit increases every year in national and foreign markets. García-Barquero and Quirós-Madrigal [4] reported that just in Costa Rica, the amount imported between 2003 and 2006 ranges between 15,985 kg and 26,885 kg, respectively. Moreover, the young stems of this plant can also be used as food [5].

Currently, some parts of this plant, such as the cladodes, have been studied to evaluate their bioactive compounds, antioxidant capacity, and potential biological activity [6]. However, phytosanitary problems have been reported recently in different types of cacti caused by insects [7,8,9,10]. *Cactophagus spinolae* Gyllenhal, 1938 (*C. spinolae*) [11] is an insect known as the “cactus borer” or “prickly pear weevil”; it is considered an endemic species to Mexico that has been reported in different states of the country and in the south of the United States [12,13,14,15,16,17]. In different studies, it has been reported that *C. spinolae* attacks the stems of *Cereus*, *Cylindropuntia*, *Ferocactus*, *Hylocereus*, *Opuntia*, *Pereskiopsis*, and *Stenocereus* genera, as well as the leaves of the genus *Agave*, belonging to the families Cactaceae and Asparagaceae [13,15,17,18,19,20]. *C. spinolae* is a pest insect that mainly attacks the *Opuntia* genus. Adults feed on the margins of young cladodes, and females lay their eggs on the basal cladodes. After hatching, the larvae consume the cladode, generating internal galleries that weaken the plant walls’ structures. In severe cases, the damage caused by this insect can compromise the plant’s survival by causing necrosis in the plant tissue [11,20]. It has been observed that *C. spinolae*, in addition to affecting *Opuntia*, also feeds on species of the *Hylocereus* genus. According to the report by Ramírez-Delgadillo et al. [13], *C. spinolae* larvae can consume up to 1% of bracts and floral buds, which can hurt the plant since the activity of this insect can extend beyond the cladodes. In recent years, it has been observed that different factors may be causing the expansion of various agricultural pests, such as climate change [21]; this could explain the transition of *C. spinolae* to new hosts like *S. undatus* in geographic locations that had not been previously reported. Vargas-Madriz et al. [20] reported the first sighting of *Cactophagus spinolae* feeding on *Selenicereus undatus* in Guerrero, Mexico, identifying *S. undatus* as a reproductive host for this weevil. Based on this precedent, the present study highlights the issue of the geographic expansion of *C. spinolae* by observing its presence in wild pitahaya populations in Jalisco, Mexico. This suggests that the pest could spread to other regions of the same country. However, from the 2023 study until the time of this research, no monitoring strategies have been implemented to control this pest. Therefore, the present study emphasizes the importance of implementing monitoring strategies to control the geographic expansion of *C. spinolae*, while also alerting the scientific community to this agronomic issue currently affecting this crop.

Pests and diseases affecting pitahaya are significant biotic factors that impact its productivity. This fruit is predominantly grown in tropical and subtropical regions, where climatic conditions favor the proliferation of pathogens, which can cause economic losses of up to 44% [22]. However, knowledge about the pests that impact pitahaya cultivation in Mexico is limited; unlike other countries such as Nicaragua [23], where more information is available, at least 12 insects associated with this crop have been reported in Mexico [24]. Among the most significant pests is *Cactophagus spinolae* [25]; however, its specific life cycle in pitahaya is unknown. Furthermore, refs. References [26,27] indicate that in *Opuntia* spp. crops, this insect has an annual generation, with active adults between May and September; furthermore, they can live up to a year in these crops.

The *C. spinolae* pest in pitahaya crops has a negative economic impact due to the damage caused to the plant and fruit, which are marketed internationally. This type of agronomic problem must be addressed through monitoring to develop appropriate pest management strategies that ensure sustainable production and prevent further infestations. This work provides a new record on the distribution of *C. spinolae* in Mexico, being the first report of this species feeding on pitahaya in the state of Jalisco, Mexico.

Currently, there is no effective methodology for the management control of *C. spinolae* in pitahaya crops. Thus, we aim to report the feeding habits and damage caused by *C. spinolae* on the vascular cylinders and flower buds of *S. undatus* in Jalisco, Mexico.

## 2. Results and Discussion

The larvae extracted from the *S. undatus* plants were yellow, 2.1 cm long, and lacked legs (legless). The collected adults measured between 1.5 and 2.5 cm in body length. Morphologically, they were characterized by the presence of two well-defined orange bands on the elytra, a broad-looking last abdominal segment, and an antennal funiculus composed of six segments. The morphological characteristics observed in the larvae and adults coincided with the description of *C. spinolae* reported by several authors [11,15,20,28].

**Figure 1:**
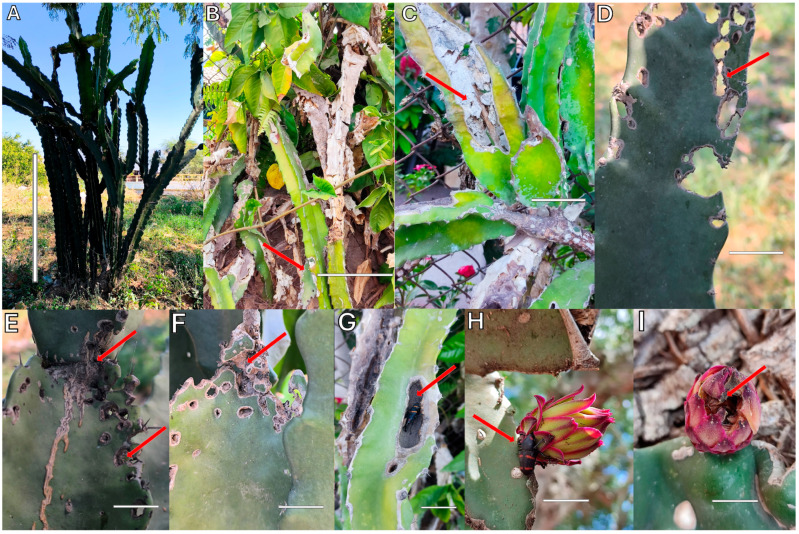
Damage caused by *Cactophagus spinolae* in *Selenicereus undatus*. (**A**) Pitahaya (*Selenicereus undatus*) plant; (**B**–**G**) damage to vascular cylinders; (**H**,**I**) damage to flower buds. Red arrows indicate the feeding or entry points of the insect; white lines highlight vascular damage. Photograph by Araceli Chino-Cantor and Haidel Vargas-Madriz.

As shown in Figure 1A, the collected specimens actively fed on *S. undatus*, causing damage to the plant, mainly in the vascular cylinder of mature and immature stems (Figure 1B–D,F,G), as well as the floral buds (Figure 1H,I). During feeding, adults perforated plant tissues, causing holes that compromised the structure and functionality of the plant. It is well documented that this type of perforation made by *C. spinolae* allows the entry of other pathogens, such as bacteria and fungi, generating indirect damage from this insect by weakening the vascular system and the absorption of water and nutrients from the plant, while increasing the probability of deterioration due to other diseases [29]. The damage observed in this study can cause negative consequences on crop yield and fruit nutritional composition [30]. The infestation of this insect in the pitahaya plant is worrying and could translate into economic losses for producers, which highlights the need to develop adequate strategies for the management of *C. spinolae* in pitahaya crops [7,10].

**Figure 2:**
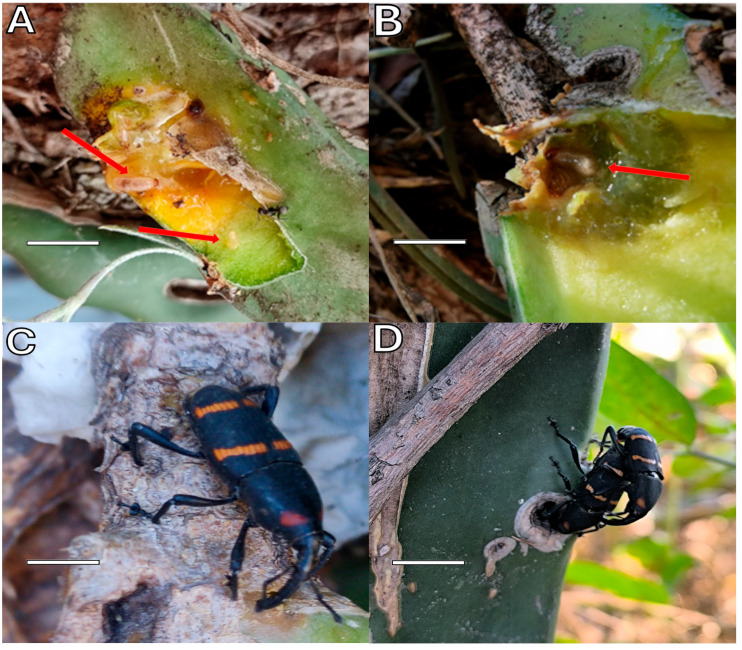
Life cycle of *Cactophagus spinolae* on *Selenicereus undatus*. (**A**,**B**) Larvae and eggs inside plant tissue. (**C**) Adult *C. spinolae*. (**D**) Copulation of adults on the plant surface. Red arrows indicate the feeding or entry points of the insect; white lines highlight vascular damage. Photograph by Araceli Chino-Cantor and Haidel Vargas-Madriz.

Monitoring also allowed for the identification of different stages in the life cycle of *C. spinolae* in *S. undatus*. The oviposition of eggs (*n* = 9) in vascular stems was observed (Figure 2A), as well as the presence of larvae (*n* = 7) within the fleshy plant tissue and larvae (*n* = 2) in mature stems (Figure 2B). Reproductive activity of adults (*n* = 16) was evident, as individuals in the feeding and copulation phase were observed on the surface of the plant during monitoring (Figure 2C,D).

This study indicates that pitahaya is not only a food source for this insect but also a potential host because the different stages of the life cycle of *S. undatus* were observed on the same plant. This is a key indicator of infestation potential, reinforcing its importance as a pest in pitahaya crops [9].

Other studies have mentioned that pests of the Curculionidae family can complete their life cycle on a single host plant, thus facilitating their spread. For example, *Diaprepes abbreviatus* completes its life cycle on a single host plant, from oviposition on the leaves to larval development in the roots [31]; this would present a phytosanitary problem in pitahaya crops. However, further studies are needed to determine whether *C. spinolae* may exhibit variations in its life cycle, fecundity, and egg hatching rate when feeding on *S. undatus* compared to its usual host plants. This information would provide valuable insights into the impact this insect has on pitahaya production and thus inform strategies for controlling this pest.

The results of this study also confirm that *C. spinolae* feeds and reproduces on *S. undatus*, causing significant damage to the stems and flower buds of the plant. These findings are consistent with previous reports documenting the association of *C. spinolae* with various cactus species in Mexico, including *Opuntia* and *Hylocereus*, indicating that the insect has a wide host range within the Cactaceae family [17]. The morphological identification of the larvae and adults collected from *S. undatus* agrees with previous descriptions of *C. spinolae*, which describe it as a weevil that affects commercial and wild cacti in Mexico [7,10]. In addition, these results expand our knowledge of the geographic distribution and host range of the pest by demonstrating its impact on *S. undatus* in Jalisco.

Potential factors that could be modifying the geographic range of hosts derive from climate change and urbanization [18,21]. Temperature changes and fluctuations in precipitation patterns and soil nutrients could also be favorable factors for the colonization of new hosts. In the case of *C. spinolae* in *S. undatus*, this could be related to these agro-environmental alterations, which is why it would be important to emphasize studies on the dispersion dynamics of this insect.

## 3. Materials and Methods

The study was carried out in an urban area of “Lagunillas” and “Autlán de Navarro”, belonging to the municipality of Autlán de Navarro, in the state of Jalisco, Mexico (19°46′17″ N, 104°21′55″ W; 900 m.a.s.l.). To characterize the infestation of *C. spinolae* in *S. undatus* (pitahaya), adults and larvae of *C. spinolae* were collected manually, extracting larvae and adults from the vascular cylinders and flower buds of the infested plants directly from pitahaya plants every 15 days from July to October 2024. The samples were placed in plastic bottles, and 70% alcohol was used as a preservative. During the sampling, visual inspections were carried out on wild pitahaya plants, recording the presence of damage symptoms attributed to *C. spinolae*, such as perforations in the cladodes and structural deterioration of the tissues.

The collected organisms were placed in polyethylene containers with 70% (*v*/*v*) ethyl alcohol for preservation. Subsequently, the samples were transported to the laboratory of the Centro Universitario de la Costa Sur of the University of Guadalajara, where their taxonomic identification was carried out. The species was determined using specialized morphological keys and comparison with reference specimens.

The taxonomic classification of the *S. undatus* plants was carried out by M. en C. Luis Guzmán Hernández, belonging to the Department of Ecology and Natural Resources of the Centro Universitario de la Costa Sur of the University of Guadalajara, Jalisco, Mexico.

## 4. Conclusions

In this research, new records were obtained on the distribution of *C. spinolae* in Mexico, documenting for the first time this species feeding on pitahaya in the state of Jalisco, Mexico. Moreover, the damage caused by this species in the different parts of *S. undatus* was reported. The results suggest the need for entomological analyses of this insect pest and its population dynamics. In addition, the examination of *C. spinolae* interactions with other organisms in the ecosystem is needed for the potential development of pest management strategies. Finally, continuous monitoring of *S. undatus* plants may be beneficial, as *C. spinolae* presence potentially represents an emerging threat to the production and quality of *S. undatus* fruits, currently of high commercial value.

## Data Availability

Additional data will be made available upon reasonable request.
